# Kawasaki Disease (KD) With Linear Facial Erythema Coinciding With Blaschko’s Lines

**DOI:** 10.7759/cureus.25568

**Published:** 2022-06-01

**Authors:** Kazuki Iio, Yu Ishida, Masaru Miura

**Affiliations:** 1 Department of Pediatrics, Tokyo Medical University Hachioji Medical Center, Tokyo, JPN; 2 Division of Pediatric Emergency Medicine, Tokyo Metropolitan Chilldren's Medical Center, Tokyo, JPN; 3 Department of Cardiology, Tokyo Metropolitan Children's Medical Center, Tokyo, JPN

**Keywords:** intravenous immunoglobulin therapy, cervical lymphadenopathy, febrile rash, blaschko’s line, kawasaki disease (kd)

## Abstract

Kawasaki disease (KD) is a self-limited, systemic vasculitis developing in early childhood. Skin findings of KD are polymorphous, varying from diffuse maculopapular eruptions to psoriasiform lesions. We described herein an 18-month-old male patient with KD who presented with linear, facial erythema coinciding with Blaschko’s lines. Parental consent for this case report was obtained in written and verbal form.

## Introduction

Kawasaki disease (KD) is a self-limited, systemic vasculitis developing in early childhood and is also known to be the leading cause of acquired heart disease in children worldwide [1]. Although the exact cause of KD is still unclear, interaction between environmental factors and polymorphisms in several susceptibility genes is considered key to its pathogenesis [1]. Since intravenous immunoglobulin (IVIG) administration within 10 days of fever onset reduces the incidence of coronary artery aneurysms from around 25% to 5% [2,3], prompt diagnosis of KD is critical. However, the diverse clinical manifestations observed in KD sometimes make this challenging because the diagnosis of KD relies solely on physical findings. Rashes are one of the five principal criteria of a KD diagnosis but show a wide range of manifestations [1]. We described herein an 18-month-old male patient with KD who presented with linear, facial erythema coinciding with Blaschko’s lines.

## Case presentation

**Figure 1:**
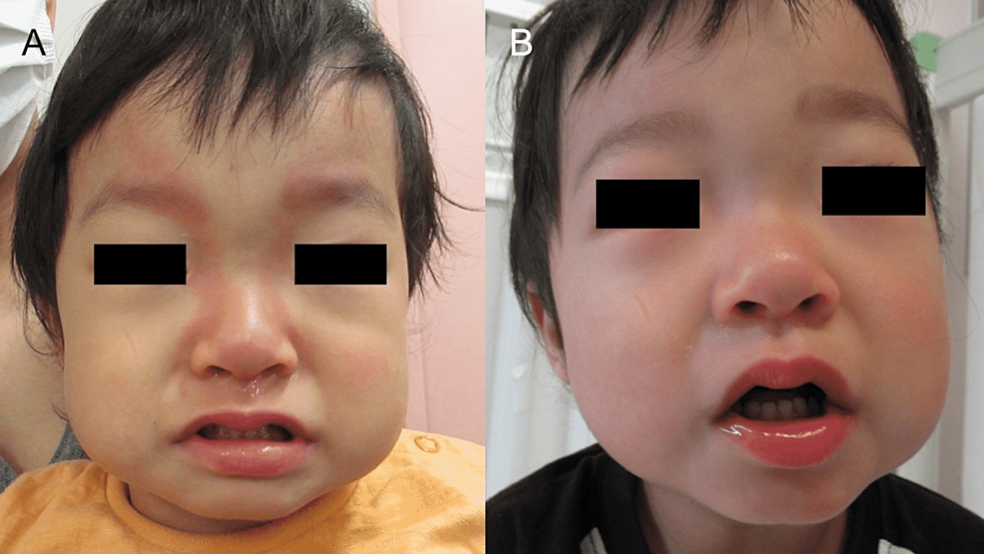
Linear, facial erythema At presentation, linear, facial erythema radiating symmetrically from the nasal root to the forehead was observed (A). The erythema resolved promptly after intravenous immunoglobulin (IVIG) administration (B).

An 18-month-old male patient presented with a four-day history of fever. He became irritable on the day of presentation. Subsequently, he presented with conjunctival injection and erythema of the lips. The appearance of linear, facial erythema simultaneously with the other findings caused considerable concern in his parents. The patient had no history of recent animal contact or travel and had been treated at our hospital for incomplete KD with IVIG when he was three months old without any cardiac complications. No skin findings occurred during his first KD episode, and he had no history of linear, facial erythema during his other fever episodes. His cousin also had a history of KD. His heart rate, respiratory rate, and body temperature were 185 beats/minute, 40/min, and 39.8℃, respectively. His oxygen saturation was 99% in room air. Physical examination revealed bilateral conjunctival injection, erythematous lips, left cervical lymphadenopathy, and erythema of the extremities, which led to a diagnosis of KD by fulfilling four of the five, cardinal, diagnostic criteria [4]. The linear, facial erythema radiated symmetrically from the nasal root to the forehead (Figure 1A), coinciding perfectly with Blaschko’s lines on the face [5]. There were no other rashes on his trunk or extremities. Laboratory tests found hyponatremia, elevated aspartate transaminase, alanine transaminase, and C-reactive protein (Table 1). SARS-CoV-2 polymerase chain reaction test was negative. There were no coronary artery lesions before the initial treatment. He was admitted and received IVIG 2 g/kg, prednisolone 2 mg/kg/day, and aspirin 30 mg/kg/day [6]. On the next day, his fever subsided to 36.5 ℃, and all the physical findings of KD, including the linear, facial erythema, resolved, leaving only mildly erythematous lips (Figure 1B). The fever did not recur, and he was discharged without any coronary artery aneurysms.

**Table 1:** Laboratory data at the presentation Laboratory data at the presentation revealed hyponatremia, elevated aspartate transaminase, alanine transaminase, and C-reactive protein.

Indicator	Unit	Patient's results	Reference range
White cell count	×/µL	6,240	4,000-8,000
Neutrophils	%	64.1	40.0-60.0
Eosinophils	%	0.5	1.0-6.0
Lymphocytes	%	22.9	25.0-45.0
Monocytes	%	12.3	3.0-7.0
Hemoglobin	g/dL	11.2	13.5-17.5
Platelet cell count	×10^4^/µL	30.4	15.0-35.0
Aspartate aminotransferase	U/L	511	13-30
Alanine aminotransferase	U/L	422	10-42
Sodium	mEq/L	134	138-145
C-reactive protein	mg/dL	11.9	0.00-0.14
Total bilirubin	mg/dL	0.7	0.4-1.5
Albumin	g/dL	3.6	4.1-5.1

## Discussion

A rash is the common manifestation of KD and occurs in 80 to 90% of patients [7]. A variety of dermatological findings, ranging from maculopapular eruptions to rare psoriasiform lesions, are known to occur in KD [1]. The present case was the first to involve erythema occurring along Blaschko’s lines. Blaschko’s lines are embryonal, epidermal cell lines distinct from the dermatome, and cutaneous lesions following Blaschko’s lines are a manifestation of cutaneous mosaicism [5]. In the present instance, the linear erythema resolved promptly in response to primary IVIG therapy as with the other KD symptoms, suggesting that its pathogenesis was linked to that of the KD.

Previous studies have reported skin lesions occurring along Blaschko’s lines in several types of polygenic inflammatory disorder, including psoriasis, lupus erythematosus, dermatomyositis, morphea, and lichen planus [8-10]. Linear skin lesions following Blaschko’s lines are thought to arise from mosaic variants in susceptibility genes, which render the affected skin areas more vulnerable to environmental irritants. KD may be another example of an inflammatory disorder that can present with skin findings following a Blaschkoid distribution.

Erythema multiforme (EM) is a major mimicker of KD and is also known to develop along Blaschko's lines [11-13]. It is an acute immune-mediated disorder characterized by cutaneous targetoid rashes and mucosal lesions [14]. Although all the previously reported EM cases with Blaschkoid lesions were of adult patients [11-13], EM-like rashes are relatively common in KD, and differentiating the symptoms may be difficult when EM develops in a febrile child [15]. To prevent delays in diagnosis, pediatricians should be careful not to overlook the non-cutaneous findings of KD whenever linear, Blaschkoid dermatitis occurs.

## Conclusions

In conclusion, patients with KD may present with linear, facial erythema distributed along Blaschko’s lines. Clinicians should be aware of the wide variety of skin findings in KD. If they are atypical, a thorough assessment for other, major signs of KD is recommended for a prompt diagnosis.
